# scROSHI: robust supervised hierarchical identification of single cells

**DOI:** 10.1093/nargab/lqad058

**Published:** 2023-06-16

**Authors:** Michael Prummer, Anne Bertolini, Lars Bosshard, Florian Barkmann, Josephine Yates, Valentina Boeva, The Tumor Profiler Consortium, Rudolf Aebersold, Melike Ak, Faisal S Al-Quaddoomi, Jonas Albinus, Ilaria Alborelli, Sonali Andani, Per-Olof Attinger, Marina Bacac, Daniel Baumhoer, Beatrice Beck-Schimmer, Niko Beerenwinkel, Christian Beisel, Lara Bernasconi, Anne Bertolini, Bernd Bodenmiller, Ximena Bonilla, Lars Bosshard, Byron Calgua, Ruben Casanova, Stéphane Chevrier, Natalia Chicherova, Maya D’Costa, Esther Danenberg, Natalie Davidson, Monica-Andreea Drăgan, Reinhard Dummer, Stefanie Engler, Martin Erkens, Katja Eschbach, Cinzia Esposito, André Fedier, Pedro Ferreira, Joanna Ficek, Anja L Frei, Bruno Frey, Sandra Goetze, Linda Grob, Gabriele Gut, Detlef Günther, Martina Haberecker, Pirmin Haeuptle, Viola Heinzelmann-Schwarz, Sylvia Herter, Rene Holtackers, Tamara Huesser, Anja Irmisch, Francis Jacob, Andrea Jacobs, Tim M Jaeger, Katharina Jahn, Alva R James, Philip M Jermann, André Kahles, Abdullah Kahraman, Viktor H Koelzer, Werner Kuebler, Jack Kuipers, Christian P Kunze, Christian Kurzeder, Kjong-Van Lehmann, Mitchell Levesque, Sebastian Lugert, Gerd Maass, Markus G Manz, Philipp Markolin, Julien Mena, Ulrike Menzel, Julian M Metzler, Nicola Miglino, Emanuela S Milani, Holger Moch, Simone Muenst, Riccardo Murri, Charlotte K Y Ng, Stefan Nicolet, Marta Nowak, Patrick G A Pedrioli, Lucas Pelkmans, Salvatore Piscuoglio, Michael Prummer, Mathilde Ritter, Christian Rommel, María L Rosano-González, Gunnar Rätsch, Natascha Santacroce, Jacobo Sarabia del Castillo, Ramona Schlenker, Petra C Schwalie, Severin Schwan, Tobias Schär, Gabriela Senti, Franziska Singer, Sujana Sivapatham, Berend Snijder, Bettina Sobottka, Vipin T Sreedharan, Stefan Stark, Daniel J Stekhoven, Alexandre P A Theocharides, Tinu M Thomas, Markus Tolnay, Vinko Tosevski, Nora C Toussaint, Mustafa A Tuncel, Marina Tusup, Audrey Van Drogen, Marcus Vetter, Tatjana Vlajnic, Sandra Weber, Walter P Weber, Rebekka Wegmann, Michael Weller, Fabian Wendt, Norbert Wey, Andreas Wicki, Mattheus H E Wildschut, Bernd Wollscheid, Shuqing Yu, Johanna Ziegler, Marc Zimmermann, Martin Zoche, Gregor Zuend, Daniel Stekhoven, Franziska Singer

**Affiliations:** Nexus Personalized Health Technologies, ETH Zurich, Zurich, Switzerland; Swiss Institute of Bioinformatics (SIB), Zurich, Switzerland; Nexus Personalized Health Technologies, ETH Zurich, Zurich, Switzerland; Swiss Institute of Bioinformatics (SIB), Zurich, Switzerland; Nexus Personalized Health Technologies, ETH Zurich, Zurich, Switzerland; Swiss Institute of Bioinformatics (SIB), Zurich, Switzerland; Institute for Machine Learning, Department of Computer Science, ETH Zurich, Zurich, Switzerland; Institute for Machine Learning, Department of Computer Science, ETH Zurich, Zurich, Switzerland; Swiss Institute of Bioinformatics (SIB), Zurich, Switzerland; Institute for Machine Learning, Department of Computer Science, ETH Zurich, Zurich, Switzerland; Cochin Institute, Inserm U1016, CNRS UMR 8104, Paris Descartes University UMR-S1016, Paris 75014, France; Swiss Institute of Bioinformatics (SIB), Zurich, Switzerland; Nexus Personalized Health Technologies, ETH Zurich, Zurich, Switzerland; Swiss Institute of Bioinformatics (SIB), Zurich, Switzerland; Nexus Personalized Health Technologies, ETH Zurich, Zurich, Switzerland; Swiss Institute of Bioinformatics (SIB), Zurich, Switzerland

## Abstract

Identifying cell types based on expression profiles is a pillar of single cell analysis. Existing machine-learning methods identify predictive features from annotated training data, which are often not available in early-stage studies. This can lead to overfitting and inferior performance when applied to new data. To address these challenges we present scROSHI, which utilizes previously obtained cell type-specific gene lists and does not require training or the existence of annotated data. By respecting the hierarchical nature of cell type relationships and assigning cells consecutively to more specialized identities, excellent prediction performance is achieved. In a benchmark based on publicly available PBMC data sets, scROSHI outperforms competing methods when training data are limited or the diversity between experiments is large.

## INTRODUCTION

After more than two decades of technological development from its earliest attempts (1,2), single cell transcriptomics studies have come of age and are widely used for basic as well as translational research (3–5). This is best showcased by the recent explosion of single cell atlases of various organs and organisms (6–9), as well as the use of single cell transcriptomics for disease investigation (10). The term ‘atlas' describes the result of identifying each and every cell type in the analyzed tissue sample for known cell types and discovering novel cell types defined by their transcriptomic phenotype. Performing such cell type annotation manually is often a labor-intensive process requiring expert field knowledge, in particular in the presence of closely related, unknown, or novel cell types such as developing or precursor cells.

In recent years, a large number of tools have been developed to automate cell type identification with varying performance, as summarized in a recent benchmark study (11). The common theme of these tools is that the expression profile of a target cell is compared to known expression profiles of particular cell types, possibly limited to a subset of genes that are relatively stable and highly expressed. In order to derive expression features predictive for a cell type, it is commonplace to use unsupervised clustering of a single cell data set and assign the cluster labels to cell types based on biological interpretation. In the next step, this cell type label is interpreted as the ground truth to build a machine learning model that finds the features relevant for cell type prediction.

However, as will be shown in the course of this work, learning features and performing the classification on the same data can lead to overfitting even if separate training and test data are used, provided they both were acquired under the same experimental condition. As a consequence, the cell type classification uncertainty is underestimated during validation and the true misclassification rate in the test situation is unexpectedly large. In other words, in practice often features (i.e. expression levels) learned in one study are applied to other studies, e.g. of the same tissue type, with the assumption that the same features enable a robust classification across studies. However, expression values can largely vary between experiments, and thus this assumption can be violated in which case features should not be used across studies. As a consequence, methods dependent on training data are again prone to an increased misclassification rate when applied to new data.

Another challenge in cell type classification is that sometimes the number of possible candidate cell types is large, which tends to increase the misclassification rate. However, mostly the cell types are related because they are the product of differentiation from a smaller number of precursor cell types.

To address these challenges, we present **s**ingle **c**ell **RO**bust **S**upervised **H**ierarchical **I**dentification of cell types (scROSHI), which utilizes a-priori defined cell type-specific gene list and does not require training or the existence of annotated data. scROSHI is independent of any information on expression levels of the cell type-specific gene lists, and thus less prone to overfitting to any particular data set. In addition, scROSHI respects the hierarchical nature of cell type relationships due to differentiation within a lineage and it assigns cells consecutively to more specialized identities. This allows to distinguish even closely related and expression-wise similar cell types. Taken together, scROSHI achieves excellent prediction performance, which we showcase by comparing the performance of scROSHI with three existing tools that scored among the best in a recent benchmark study (11). To capture a realistic scenario, we utilize three annotated datasets and apply methods across those data, i.e. cell typing is performed on a dataset different from the training set. We show that scROSHI outperforms the competing methods when the training dataset differs from the data that is evaluated for cell typing.

Taken together, scROSHI is a transparent, interpretable, and robust cell type classification approach particularly useful when previous knowledge about cell type-specific genes is available but annotated training data is scarce. scROSHI is available as an R package and can thus be seamlessly integrated into single cell analysis workflows.

## MATERIALS AND METHODS

The key idea of scROSHI is conceptually simple: it requires a list of cell types expected in a sample, and for each of those cell types a list of genes expected to be cell-type specific (or in the minimum prominently expressed in only one cell type). Based on this information, for each cell and for each cell type scROSHI compares the expression of the cell type-specific genes with the expression of the genes selected for the other expected cell types. The assumption is that a single cell is 100% pure, i.e. is identified by one cell type or another but not a mixture. Then each cell should show high expression of cell type-specific genes for only one cell type, which will be the cell type classified by scROSHI. Provided the observed object is indeed a single cell, this assumption can be violated in two scenarios: (i) the cell type of the cell is not among the list of presented cell types (or the quality of the cell type-specific genes is poor) and (ii) the genes of more than one cell type show high expression (e.g. because two cell types are highly similar). In the first scenario scROSHI will label the respective cell as ‘cell type unknown’ to indicate that either a novel (‘unknown’) cell type is present and/or further investigation is warranted. In the second scenario scROSHI will label the cell as ‘cell type uncertain’, again indicating the need for further investigation, while providing information of which cell types are likely candidates for classification to ease the manual interpretation.

### scROSHI: design considerations

The most important design criterion for scROSHI was that the method should be capable of automated classification in the absence of labeled training data. This excluded any machine learning approach that would require training a model. Instead, the method should utilize and rely on the vast amount of validated cell type-specific gene lists available from previous bulk or single cell experiments, such as the widely acknowledged immune cell type gene lists (also known as ‘*lm22*’) used by the cibersort algorithm (12) or the somewhat related resource for single cell melanoma data (13).

Another important design criterion was that the method should avoid re-training on a dataset currently under investigation. While, in general, inclusion of training data from a variety of sources is advantageous, at this stage, re-training would lead to overfitting and therefore to an overestimation of the prediction performance. Provided the originally chosen gene list was previously validated to be robust against changes in the experimental condition, re-training is not necessary.

This argument can be turned around to provide a strategy on how to arrive at a suitable gene list for cell types for which a previously validated set is not available: included as cell type specific genes, i.e. genes that are highly expressed in the target cell type, should be those genes that have this property independent of tissue type, sample type (i.e. cultured immortalized cells, cultured primary cells, tissue biopsy), detailed setup (culture medium, organism), or patient characteristics (gender, ethnicity, age). The larger the diversity of the test data, the more robust and broadly applicable the final gene list.

Given a set of cell type-specific genes and without the need for training a model, one possibility to assign a cell type from a list of candidates to a target cell is to test for association and choose the one that fits best. On one end of the spectrum when measuring association is the hypergeometric test comparing the proportion of highly expressed genes specific for one cell type with the proportion of highly expressed genes specific for all other cell types. The advantage is that it can almost always be calculated and is robust against expression outliers. On the one hand, it is simple to use because the cell type-specific reference does not have to be known quantitatively in the form of an expression profile. On the other hand, it is relatively insensitive because it completely ignores the quantitative nature of the expression profile of the target cell, which is typically available. On the other end of the spectrum when measuring association one can quantitatively match the expression profile of a known cell type to the expression profile of the target cell, for instance, using Spearman's correlation. While this approach is robust against expression outliers, it is relatively expensive in data availability, i.e. it requires knowledge of the gene expression profile of the reference. We chose to follow an intermediate path by performing a quantitative and robust test, the Mann-Whitney rank sum test, to compare the expression ranks of the genes specific for one cell type with the ranks of the genes specific for all other cell types. The negative log of the test's p-value is then a measure of the association strength between the target cell and this cell type. It can be interpreted as a score for how well the target cell matches the cell type at hand.

Like in most classification problems it is assumed here that each cell belongs to exactly one class of a given set of candidates. However, scROSHI goes one step further and allows for the introduction of two additional classes, *unknown* and *uncertain*, to deal with the unavoidable classification uncertainty.

Another step to improve the classification efficiency is to utilize the hierarchical tree structure that is inherent to cell types due to developmental specialization and therefore apply a hierarchical classification approach. Instead of classifying all cell types at once, target cells are first assigned to a smaller number of major cell types and then consecutively to more specialized classes. This way, relatively similar cell types can be distinguished provided they belong to different branches in the tree.

### The scROSHI workflow

1. Find out which cell types to expect from field knowledge.
2. Obtain validated cell type-specific gene lists from the literature or learn cell type specific genes based on other datasets. Importantly, only the gene names, not the expression, is relevant.
3. Optional: Obtain a hierarchical tree structure to define cell type parent–kin relationships.
4. For each cell $i$ and each cell type $j$ in the first hierarchical level, compare the expression of the genes specific for this cell type with the expression of the genes selected for the other expected cell types: determine the *P*-value of a one-sided Mann-Whitney test of the Null hypothesis that the expression rank sum of the genes specific for this cell type $j$ is the same as or smaller than the rank sum of the genes specific for any other cell type in the list.
  $$\begin{equation*}H_0^{ij}:\mathop \sum \limits_{{g}_k} Rank( {{g}_{k\ = \ j}} ) \le \mathop \sum \limits_{{g}_k} Rank( {{g}_{k \ne j}} )\end{equation*}$$
  The alternative hypothesis is that the rank sum of the genes specific for this cell type is larger.
5. Compute the normalized negative log of the *P*-value for each cell $i$ and each cell type $j$, respectively.
  $$\begin{equation*}{s}_{ij} = \frac{{ - {{\log }}_{10}( {{p}_{ij}} )}}{{\mathop \sum \nolimits_{ij} - {{\log }}_{10}({p}_{ij})}}\end{equation*}$$
  Interpret the result as a score for how well the cell matches that cell type.
6. Assign the cell type label with the highest score to the cell.
7. If none of the scores is above a certain threshold, do not assign a cell type label to the cell but assign it to the class ‘unknown’.
8. If the ratio between the largest and the second largest score is below a certain threshold, do not assign a cell type label to the cell but assign it to the class ‘uncertain’.
9. Repeat 4 to 7 for the second hierarchical level, and so on. Cells that have been classified as ‘unknown’ or ‘uncertain’ in the first iteration are included in the next iteration to allow classification into next level cell types.

**Figure 1.**
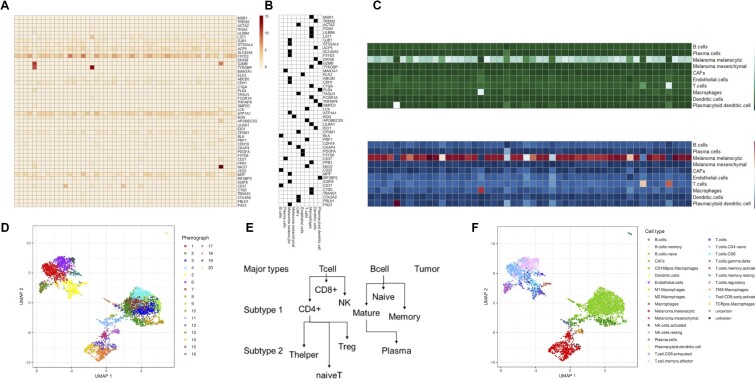
Schematics of the scROSHI workflow. The gene x cell (row x column) normalized expression matrix (**A**) is combined with the binary gene × cell type membership matrix (**B**) to define genes specific for a cell type (black) and genes specific for other cell types (white). (**C**) The one-sided Mann-Whitney rank sum test provides a cell type x cell score matrix (top), which is normalized (bottom). (**D**) UMAP representation of all cells from a melanoma patient biopsy using the most highly variable genes, colored by phenograph clusters. (**E**) Developmental ‘family tree’ defining cell type hierarchies. (**F**) The representation in (D) is colored by scROSHI predicted cell types.

scROSHI takes as input the gene x cell count matrix, either with raw or normalized counts (Figure 1A). scROSHI is robust to the choice of normalization and/or transformation method, because the cell type score is based on ranks rather than on the actual values. In our studies we typically use sctransform (14), which corrects unwanted biases using regularized negative binomial regression. In general, it is advised that the scale of the input data matches the scale at which the cell type-specific gene lists were generated.

The second ingredient required for cell type classification is a collection of cell type-specific gene lists (Figure 1B). The selection of cell types to expect will depend on the nature of the sample. It is recommended to adapt the cell type selection to keep classification specificity high whenever possible: having closely related cell types in the candidate list may sometimes be required but should be avoided if possible.

There are a number of resources available containing curated reference datasets, mostly assembled from bulk RNA-seq or microarray data of sorted cell types. Examples are the C8 set of the MSigDB collection (15), the lm22 immune cell list of cibersort (12), the BioGPS Human Cell Type and Tissue Gene Expression Profiles collection (16) from harmonizome (17), or the Bioconductor (18) package celldex (19). These references are often good enough for most applications provided that they contain the cell types that are expected to be present in the data at hand. For our contribution to the Tumor Profiler Study (20), working on melanoma patient biopsy samples, we used the curated gene list from Tirosh et al.(13) in combination with the immune cell list of cibersort.

In cases where quantitative cell type-specific reference profiles are available they can be used as is or they can be binarized to obtain cell type-specific gene lists (Figure 1B). They should contain genes that show little variability and are highly expressed in the target cell type and have zero or weak expression in all other cell types. The gene lists do not need to be exclusive, i.e. the same gene can appear in different cell type lists, but the overlap between cell types should be kept small. Obviously, the more similar two cell types are, the larger the overlap between their specific gene lists will be. In addition, given the sparsity of single cell count data, gene lists with only a few members will have lower sensitivity compared to larger lists.

The third ingredient to scROSHI is a hierarchical tree structure defining parent - kin relationships between cell types. The purpose of this tree is to classify cells first into a small number of coarse-grained cell type super-families and then consecutively into more and more specialized, fine-grained cell (sub-)types (Figure 1E). This way, the number of possible candidate cell types in each step is much smaller than the total number of candidate cell types thus reducing the possibility of false classification. Moreover, the thresholds for unknown and uncertain classes can be chosen to fit the detailed cell type similarity distribution in each branch to optimize classification efficiency.

With the three inputs (i) count matrix, (ii) cell type-specific gene lists and (iii) hierarchical relationships between cell types, scROSHI performs the cell type score assignment and classification. For each cell and each cell type, a one-sided Mann-Whitney U-test is performed. The Null hypothesis is that the expression rank sum of the genes specific for this cell type is the same as or smaller than the rank sum of the genes specific for any other cell type in the list. The alternative hypothesis is that the rank sum of the genes specific for this cell type is larger. The normalized negative log of the p-value for each cell-cell type pair is interpreted as a score how well the cell matches the respective cell type (Figure 1C). If none of the scores is above a certain threshold, no cell type label is assigned to the cell but the class ‘unknown’. Also, if the ratio between the largest and the second largest score is below a certain threshold, again no cell type label is assigned to the cell but the class ‘uncertain’. Both categories, ‘unknown’ and ‘uncertain’, can reflect populations that are not included in the list of *a priori* selected cell types, thus potentially indicating ‘novel’ cell types (or poor quality of the cell type-specific gene lists). These two categories therefore help to avoid misclassification by explicitly considering classification uncertainty and moreover point out cell populations that require further investigation. The choice of the two thresholds can be made *ad hoc* based on visual inspection of the results or consistency with other methods for unlabeled data, or based on an optimization scheme by minimizing the classification cross-entropy when ground truth-labeled data is available. In general, the higher the difference between the cell types, the more stringent can the thresholds be chosen.

Taken together, these steps facilitate an enrichment of the pure data-driven description of the single-cell data (Figure 1D) with biological meaning (Figure 1F).

### Benchmarking

A detailed description of the datasets used, their origin, which preprocessing steps were applied, as well as the description of the pipeline and the competing tools is described in the Supplementary Material (Supplementary Tables S1–S5, Supplementary Figures S1–S3).

To briefly summarize, we used public datasets with a similar cell type composition to benchmark scROSHI against high profile competitor methods. Data from three peripheral blood mononuclear cell experiments were retrieved, one from an adult human in which the cell types were pre-sorted (Zheng_sorted set), and one each of an adult (Adult set) and a newborn (Newborn set). Hence, the three sets are similar in content but differ in experimental setting and donor age.

We defined a common set of matching cell type labels across the three datasets for comparisons between datasets (see Supplementary Material for further details on the ground truth dictionary).

Based on a previous benchmark of automatic cell identification methods (11), we decided to compare scROSHI to three front runners: support vector machine (SVM), random forest (RF) and GARNETT (21). The main difference between scROSHI and its competitors is the fact that they use part of the data to train a model whereas with scROSHI there is no training involved once the cell type-specific gene lists are selected. While SVM and RF can capture non-linear relationships between the explanatory features (gene expression) as well as interactions between them, GARNETT is based on a penalized multivariate generalized linear prediction model (GLMNET). All methods, including scROSHI, were used under standard conditions, with default parameter settings.

We trained a model for RF, SVM, and GARNETT and evaluated the performance of the classifiers by applying a 5-fold cross-validation for each dataset. The folds were split in a stratified manner in order to keep equal proportions of each cell population in each fold. We used the same training and testing folds for all classifiers. scROSHI and Garnett require a cell type specific gene list. We used a list of cell type specific genes based on previous publications (12,22) for scROSHI and a garnett-optimized marker list (check_markers() function from the garnett package v.0.2.17) for Garnett. In addition, the following criteria were supplied to scROSHI for classifying a cell as *unknown* or *uncertain*. A cell is labeled *unknown* if none of the *P*-values is below 0.05 and *uncertain* if the ratio between the smallest and the second smallest *P*-values is above 0.1 (major cell type) or 0.8 (subtype). These thresholds were chosen as the default settings when designing scROSHI in the context of profiling tumor samples from the Tumor Profiler study (20).

#### Validation scheme

Each of the three datasets was split in training, validation, and testing sets. Three major validation runs were performed in which each of the three datasets served as the training/validation set. After the final model was obtained, it was tested once ‘in set’ on the testing set that came from the same experiment as the training data, and two times ‘out of set’ on the two remaining sets from which the model has not yet seen any data. scROSHI was tested by the same scheme. Further details on the validation scheme can be found in the Supplementary Material.

### Copy number variation estimation

To pre-process scRNA-seq data from the Tumor Profiler Study, we used a procedure based on standard quality control measures (23). First, to retain only high quality cells, we removed cells with fewer than 700 expressed genes and 1500 total read counts detected. Second, to avoid contamination by dying cells while retaining as many informative cells as possible, we filtered out cells with more than 35% of read counts coming from mitochondrial genes (24,25).

To distinguish normal from malignant cells, we inferred large-scale copy number variations (CNVs) from the gene expression data using *infercnvpy* (https://github.com/icbi-lab/infercnvpy). We ran *infercnvpy* on every sample individually using T cells, B cells, Endothelial cells and Macrophages as reference cells. The gene ordering file containing the chromosomal start and end position for each gene was generated from the human GRCh37 assembly. To reduce the noise level, we only used genes that had a mean read count greater than 0.1.

We then used an approach based on hierarchical clustering of single cell copy number profiles to detect cells with and without CNVs. After calling CNVs, we used *scipy*’s implementation of hierarchical clustering with Ward linkage (26) to obtain a dendrogram of the CNV profiles. By definition, each node in a dendrogram only had two child nodes that represented a cluster of clusters, except for leaf nodes that represented a cluster of cells. Each cell was annotated as malignant or non-malignant using scROSHI’s cell type annotations. Starting at the root node, we then iteratively assigned a CNV status to the nodes according to the composition of their subtrees. Specifically, a node and all nodes in its subtree were annotated as presenting no CNVs if both its subtrees contained at least 60% of non-malignant cells. We traversed the dendrogram until we reached all nodes or a maximum depth of five in the dendrogram. Finally, a cell was assigned the ‘no CNVs’ status if it belonged to a leaf node that had been annotated as not presenting CNVs. All remaining cells were annotated as showing CNVs.

## RESULTS AND DISCUSSION

### Performance evaluation

We compared the performance of scROSHI on test datasets with the performance of supervised methods that had been trained with the test dataset (intra-dataset evaluation) and that had been trained with a different dataset (inter-dataset evaluation). There were three types of classifiers: (1) prior knowledge method (scROSHI) for which a cell type specific gene list is required. (2) Supervised methods (RF, SVM), which require a training dataset labeled with corresponding cell labels. (3) Combined method (GARNETT), which requires both a cell type specific gene list and a training dataset. We calculated the percentage of unlabeled cells across all cell populations per classifier. Further, we calculated the accuracy of only major cell types for scROSHI and GARNETT, since both methods perform a hierarchical cell typing with major and subtype labels (Supplementary Table S5). Additionally, we determined the proportions of cells that only have a major cell type label, cells that have label ‘unknown’, or are unclassified.

**Figure 2.**
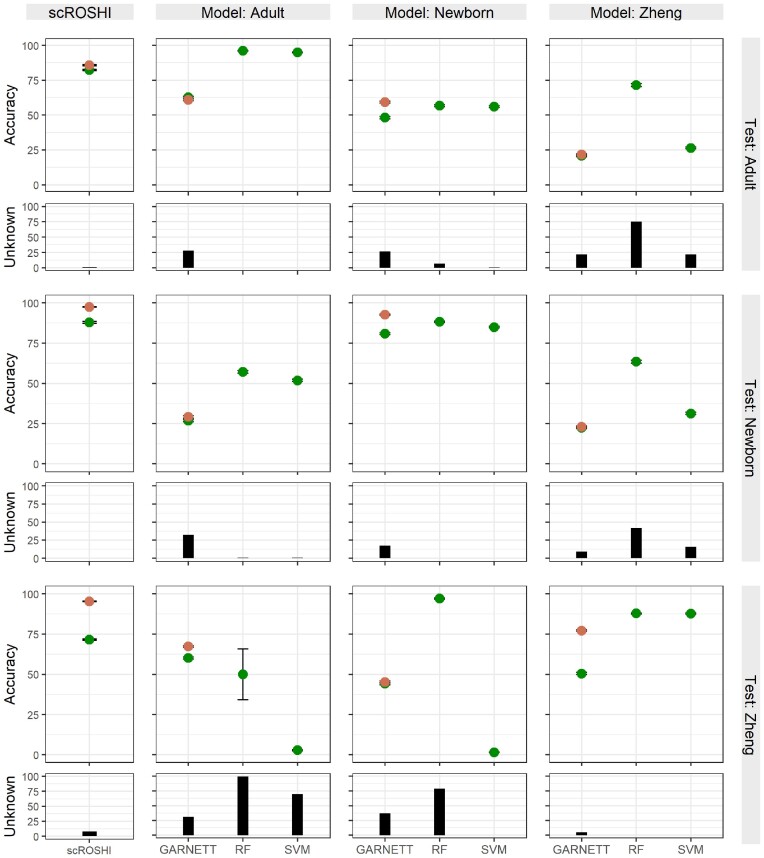
Benchmark results for scROSHI (left) and the three competing machine learning methods. Each panel corresponds to a combination of training data (column) and test data (row). The cross-validation accuracy (in%) is shown separately for major cell types (orange) and all fine-grained cell types (green). The black bar shows the percentage of cases where the cell type is ‘unknown’.

Figure 2 shows the overall results of the inter and intra-dataset evaluation. Generally, scROSHI performs as well as the supervised methods if the supervised methods were trained with the test dataset (scROSHI accuracy: adult 0.823, newborn 0.879, Zheng 0.715). However, scROSHI outperforms the supervised methods if they were trained with another dataset—in this case we observed a lower accuracy and/or a higher amount of unlabeled cells for all supervised methods, a consequence of overfitting to the training data. The supervised methods perform better if they were trained with a dataset that is closer to the test dataset (e.g. training data: Adult; test data: Newborn) but there is a strong decrease in performance if the test data is dissimilar (e.g. training data: Adult; test data: Zheng).

The subtype classification on the Zheng dataset was challenging for all classifiers (scROSHI accuracy: 0.715). However, the accuracy of the major cell type label was 0.952 for scROSHI indicating that even if it was not possible to find the correct subtype label the correct major cell type label could usually be determined. Moreover, even though the fraction of unknown cells was slightly increased for scROSHI in the Zheng dataset, considerably elevated levels were observed for the ML-based methods regardless of whether the Zheng data was involved in training or testing (Figure 2, black bars in the right column and the bottom row, respectively).

There were two cases where on first glance the out-of-training performance of the RF model was comparable (training Zheng, test Adult) or even better (training Newborn, test Zheng) than scROSHI. However, both were accompanied by an unknown cell fraction of more than 70% in RF but below 10% in scROSHI. Essentially, the high apparent accuracy was therefore only achieved at the cost of a large proportion of cells that could not be assigned any label.

All in all, our benchmark study shows that scROSHI performs superior to competing tools provided a good quality cell type specific gene list is available and annotated training data are limited or not available, which is often a realistic scenario in early stage projects. Moreover, the good performance is achieved with very reasonable amount of resources. For example, it took less than 35 s to classify 2000 cells expressing 3368 genes into 11 cell types using 6 GB RAM on a standard laptop (i7 Intel processor). And because classification is done independently cell-by-cell, even extremely large datasets can be handled by splitting into smaller batches.

Similar to the scoring tool ucell (27), the cell type score of scROSHI depends only on the relative rank of the gene expression signal, does not require normalization, and makes no assumptions about the distribution of the signal. But, because scROSHI utilizes the hierarchical nature of cell identities, it can outperform its competitor when a sample contains similar cell types that derive from different branches of the lineage tree.

scROSHI was developed for 10xGenomics mRNAseq data of tumor patient samples but there is no known limitation to use it on any other modality or organism. However, it is ideal if the cell type-specific gene lists were defined from results of the same technology as the data at hand.

One possibility to improve the performance of the machine learning tools, i.e. the accuracy on unseen data, might be to train them on a more diverse data set. Yet, because training on accuracy does not learn causal features for cell type identity, this approach by design does not lead to a universally applicable model and the performance will still be lower on unseen data than in the validation set, due to overfitting.

The hierarchical scheme in scROSHI, to successively classify cells first into more coarsely grained parent cell types, followed by more and more fine-grained sibling cell subtypes within each parent cell type, reduces the classification complexity in each branch of the tree, potentially reducing the classification error rate in turn. Moreover, the thresholds for unknown and uncertain classes can be tailored to the detailed cell type similarity distribution or count matrix sparsity within each branch.

### Consistency with estimations of copy number alterations

In addition to these benchmark datasets with known ground truth but relatively simple cell type composition we used scROSHI for cell type identification in clinical samples, i.e. biopsies from melanoma patients participating in the Tumor Profiler Study (20). In these samples the cell type composition can vary considerably from patient to patient depending, for instance, on the biopsy location, and is more complex to start with. No ground truth was available for such clinical samples, thus we evaluated the classification results by comparison to single cell CyTOF cell type composition analysis on the same samples (28) and by consistency with copy number variation (CNV) estimations (Figure 2). The rationale is that only tumor cells are expected to harbor any CNVs, and thus CNVs can be used to distinguish tumor cells from non-tumor cells.

**Figure 3.**
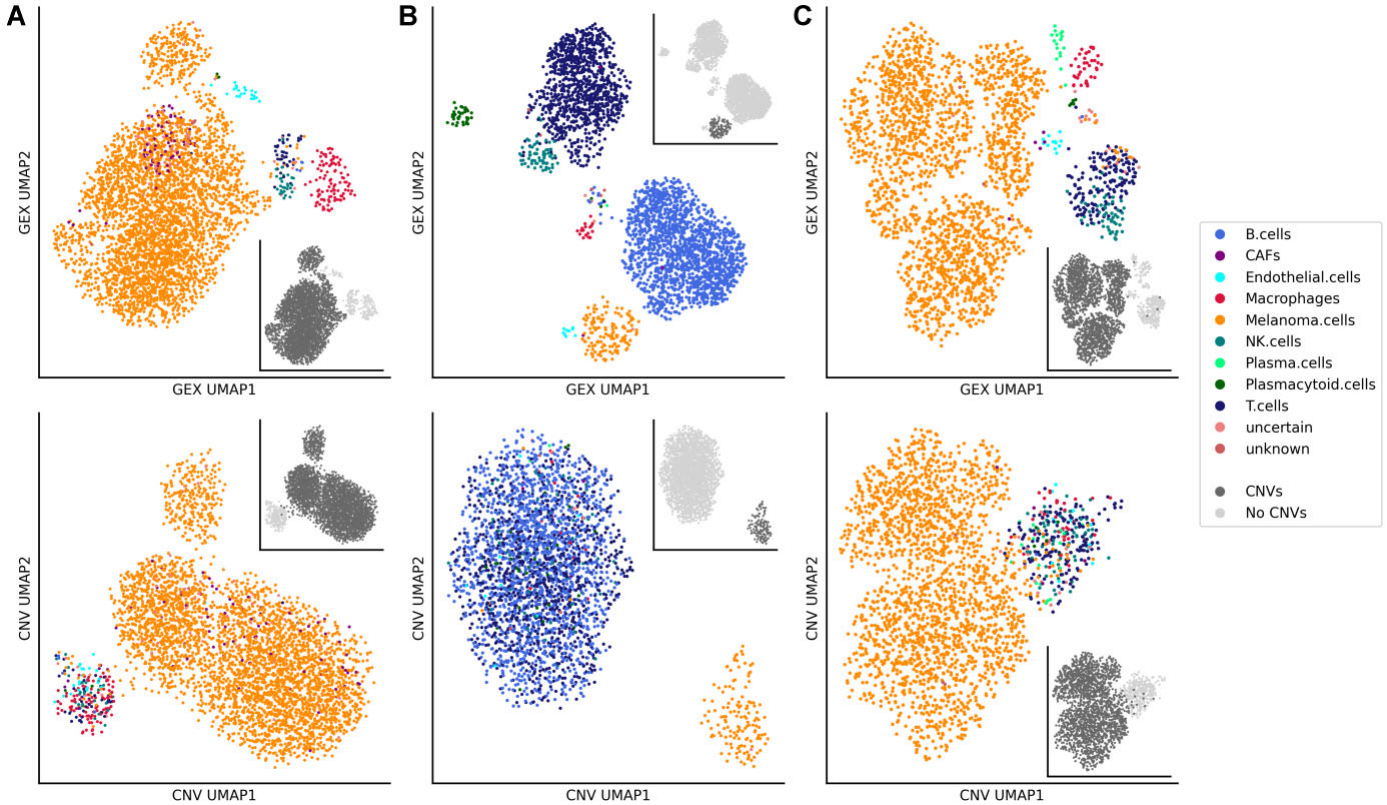
UMAP representation of cells in gene expression and CNV space. Cells from three melanoma biopsy samples (**A**, *n* = 3928 cells; **B**, n = 2967 cells; **C**, *n* = 2326 cells) were annotated using scROSHI. The first row shows the UMAP embedding of the normalized and log-transformed gene expressions and the second row shows the UMAP embedding of CNV profiles. The colors represent the cell type annotations. The greyscale in the insets represent the CNV status.

The three representative samples in Figure 3A–C show a diverse cell type composition, as illustrated by the two-dimensional UMAP representation based on gene expression in the top row. CNV states appear nearly exclusively in cells identified as melanoma cells, the only malignant cells present (insets). In Figure 3 bottom row, the focus is shifted to UMAP representations based on CNV states, where all non-malignant cells form a single cluster and malignant cells one or more separate clusters. In the sample shown in Figure 3A, a few cells located in the melanoma cluster are mis-classified as cancer associated fibroblasts (CAFs, filled purple circles), possibly a consequence of an increased copy number in melanoma cells located at some CAF specific genes and/or copy number decrease in some melanoma specific

genes. The cell type composition in these samples is dominated by melanoma cells (A: 92%, B: 8%, C: 87%), B cells (A: 0%, B: 52%, C: 1%) and T cells (A: 1%, B: 33%, C: 7%). A comparison to single cell CyTOF experiments of the same samples gave similar proportions (S. Chevrier, private communication, manuscript in preparation): melanoma cells (A: 81%, B: 2%, C: 84%), B cells (A: 0.7%, B: 40%, C: 2%) and T cells (A: 5%, B: 40%, C: 9%).

### Unexpected cell types

As we have introduced the label ‘unknown’ into scROSHI when none of the classification scores of the list of candidate cell types was high, we investigated whether this would empower scROSHI to recognize that there is an unexpected cell type present in a sample.

**Figure 4.**
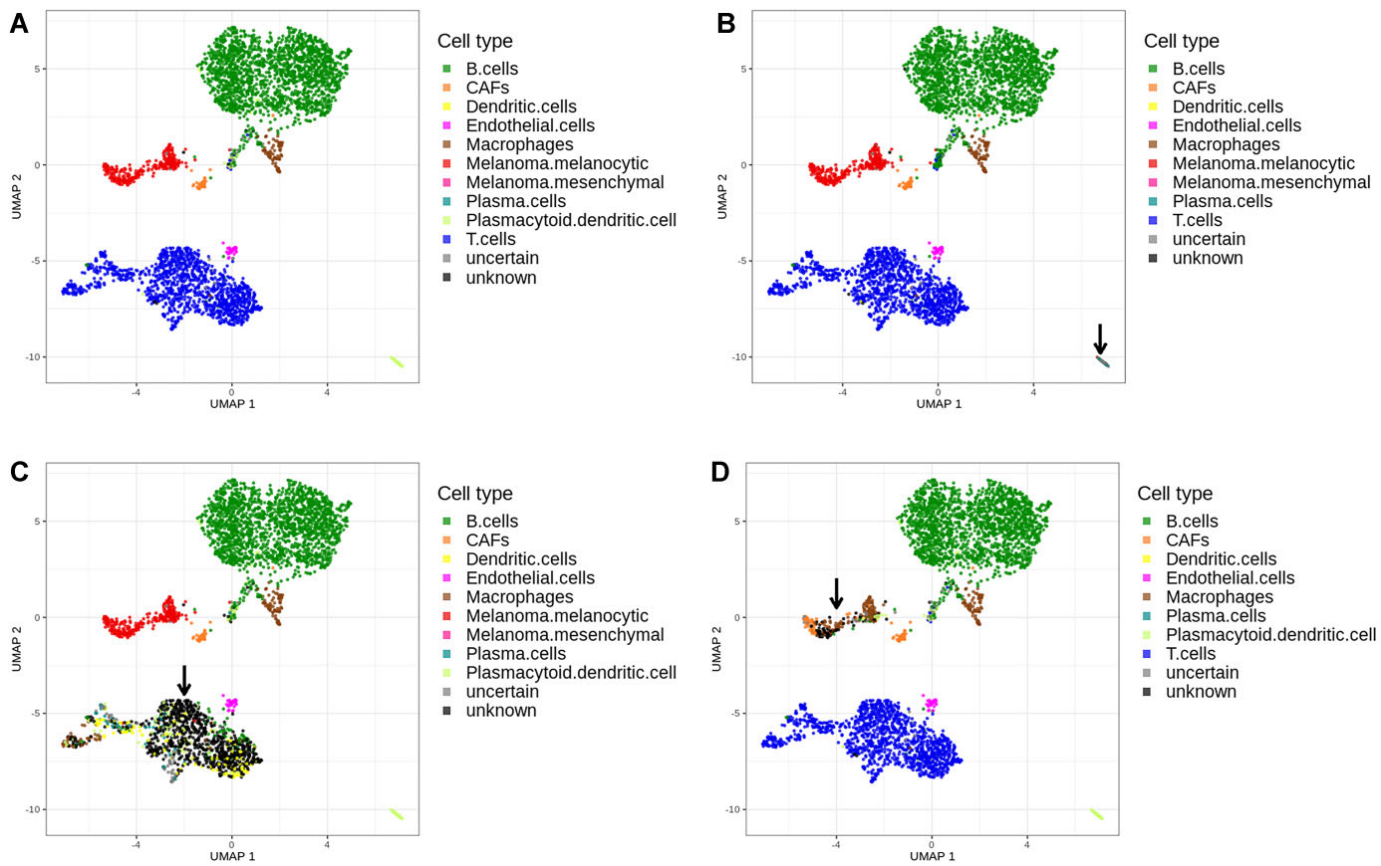
Cell type classification where the list of expected cell types was modified. (**A**) All known cell types are included. (**B**) Plasmacytoid dendritic cells are excluded. (**C**) T cells are excluded. (**D**) Melanoma cells are excluded. In panels B–D, the cluster of cells for which the label was excluded in the classification is marked by an arrow.

We simulated the situation that there is an unknown cell type in a sample by removing one cell type from the candidate cell type list. As a starting point, we used a sample from the Tumor Profiler Study (20) with several different cell types that could be identified (Figure 4A). Then we removed the genes specific for three cell types (Plasmacytoid dendritic cells (pDC), T cells, Melanoma cells) and repeated the analysis for each case (Figure 4B–D). All previously identified pDCs were classified as ‘unknown’ when excluded from the candidate list, as expected (Figure 4B, bottom right corner).

When T cells were missing in the candidate list, a small proportion was mis-classified as dendritic cells or plasma cells but the vast majority was correctly labeled ‘unknown’ (Figure 4C). Moreover, the cells now (mis-) labeled as dendritic cells are sparsely scattered across the entire former T cell population rather than forming a compact cluster or region, which would be expected if they belong to a well-defined cell type or subtype. This observation should raise suspicion and trigger further investigation as it reflects the possibility that the cell type-specific genes do not represent the profile of a distinct cell population observed in this particular study.

In contrast, when melanoma cells were removed from the candidate list, a considerable proportion was mis-labeled (Figure 4D). One particular subpopulation on the left hand side of the melanoma cluster appears to share expression features with cancer associated fibroblasts (CAFs), whereas another subpopulation on the right hand side of the melanoma cluster seems to share some similarity with macrophages. At the same time the relatively large proportion of ‘unknown’ cells in the center of the cluster indicates that the cell type candidate list is incomplete or otherwise not suitable for this kind of data. A possible explanation for this observation may be the fact that tumor cells can share expression features with other cell types by exploiting cellular plasticity and de-differentiation programs (29).

To summarize this part, most of the cells for which the cell type specifics were excluded from the candidate list were labeled as ‘unknown’ while a small proportion was misclassified. This procedure outlines how scROSHI may serve as a tool to detect novel cell types that were not expected to be in the sample under investigation.

## CONCLUSION

Cell type identification is a critical, yet challenging, step in single cell transcriptomics analysis. Although various machine learning based methods for cell typing are available, the necessity to learn features on adequate training data is prone to overfitting and also challenging in practice, in particular for studies on novel experimental conditions. Here, we have presented scROSHI, a novel supervised cell type classification method independent of training data but instead based on *a priori* defined cell type cell type specific genes. In a benchmark study and on clinical data from tumor samples, we have shown that scROSHI is useful, robust, versatile, and competitive to existing methods under real-life scenarios.

## DATA AVAILABILITY

Availability of benchmark data: ask at scp-support@broadinstitute.zendesk.com.

The three data sets from the TumorProfiler Study are available upon request at info@tu-pro.ch, and according to the data sharing policy at the web site https://eth-nexus.github.io/tu-pro_website/data/ (in preparation by the consortium). In the meantime, we have posted the raw count matrices on Zenodo (https://doi.org/10.5281/zenodo.6577402).

Code availability: scROSHI is available as R package on CRAN (https://cran.r-project.org/package=scROSHI).

## Supplementary Material

lqad058_Supplemental_File

## APPENDIX

### Membership of the Tumor Profiler consortium

Rudolf Aebersold^2^, Melike Ak^27^, Faisal S Al-Quaddoomi^9,16^, Jonas Albinus^7^, Ilaria Alborelli^23^, Sonali Andani^6,16,25,30^, Per-Olof Attinger^11^, Marina Bacac^15^, Daniel Baumhoer^23^, Beatrice Beck-Schimmer^38^, Niko Beerenwinkel^4,16^, Christian Beisel^4^, Lara Bernasconi^26^, Anne Bertolini^9,16^, Bernd Bodenmiller^8,34^, Ximena Bonilla^6,16,25^, Lars Bosshard^9,16^, Byron Calgua^23^, Ruben Casanova^34^, Stéphane Chevrier^34^, Natalia Chicherova^9,16^, Maya D’Costa^10^, Esther Danenberg^36^, Natalie Davidson^6,16,25^, Monica-Andreea Drăgan^4^, Reinhard Dummer^27^, Stefanie Engler^34^, Martin Erkens^13^, Katja Eschbach^4^, Cinzia Esposito^36^, André Fedier^17^, Pedro Ferreira^4^, Joanna Ficek^6,16,25^, Anja L Frei^30^, Bruno Frey^12^, Sandra Goetze^7^, Linda Grob^9,16^, Gabriele Gut^36^, Detlef Günther^5^, Martina Haberecker^30^, Pirmin Haeuptle^1^, Viola Heinzelmann-Schwarz^17,22^, Sylvia Herter^15^, Rene Holtackers^36^, Tamara Huesser^15^, Anja Irmisch^27^, Francis Jacob^17^, Andrea Jacobs^34^, Tim M Jaeger^11^, Katharina Jahn^4^, Alva R James^6,16,25^, Philip M Jermann^23^, André Kahles^6,16,25^, Abdullah Kahraman^16,30^, Viktor H Koelzer^30^, Werner Kuebler^24^, Jack Kuipers^4,16^, Christian P Kunze^21^, Christian Kurzeder^20^, Kjong-Van Lehmann^6,16,25^, Mitchell Levesque^27^, Sebastian Lugert^10^, Gerd Maass^12^, Markus G Manz^29^, Philipp Markolin^6,16,25^, Julien Mena^2^, Ulrike Menzel^4^, Julian M Metzler^28^, Nicola Miglino^1^, Emanuela S Milani^7^, Holger Moch^30^, Simone Muenst^23^, Riccardo Murri^37^, Charlotte KY Ng^23,33^, Stefan Nicolet^23^, Marta Nowak^30^, Patrick GA Pedrioli^3^, Lucas Pelkmans^36^, Salvatore Piscuoglio^17,23^, Michael Prummer^9,16^, Mathilde Ritter^17^, Christian Rommel^13^, María L Rosano-González^9,16^, Gunnar Rätsch^3,6,16,25^, Natascha Santacroce^4^, Jacobo Sarabia del Castillo^36^, Ramona Schlenker^14^, Petra C Schwalie^13^, Severin Schwan^11^, Tobias Schär^4^, Gabriela Senti^26^, Franziska Singer^9,16^, Sujana Sivapatham^34^, Berend Snijder^2,16^, Bettina Sobottka^30^, Vipin T Sreedharan^9,16^, Stefan Stark^6,16,25^, Daniel J Stekhoven^9,16^, Alexandre PA Theocharides^29^, Tinu M Thomas^6,16,25^, Markus Tolnay^23^, Vinko Tosevski^15^, Nora C Toussaint^9,16^, Mustafa A Tuncel^4,16^, Marina Tusup^27^, Audrey Van Drogen^7^, Marcus Vetter^19^, Tatjana Vlajnic^23^, Sandra Weber^26^, Walter P Weber^18^, Rebekka Wegmann^2^, Michael Weller^32^, Fabian Wendt^7^, Norbert Wey^30^, Andreas Wicki^29,35^, Mattheus HE Wildschut^2,29^, Bernd Wollscheid^7^, Shuqing Yu^9,16^, Johanna Ziegler^27^, Marc Zimmermann^6,16,25^, Martin Zoche^30^, Gregor Zuend^31^

^1^Cantonal Hospital Baselland, Medical University Clinic, Rheinstrasse 26, 4410 Liestal, Switzerland, ^2^ETH Zurich, Department of Biology, Institute of Molecular Systems Biology, Otto-Stern-Weg 3, 8093 Zurich, Switzerland, ^3^ETH Zurich, Department of Biology, Wolfgang-Pauli-Strasse 27, 8093 Zurich, Switzerland, ^4^ETH Zurich, Department of Biosystems Science and Engineering, Mattenstrasse 26, 4058 Basel, Switzerland, ^5^ETH Zurich, Department of Chemistry and Applied Biosciences, Vladimir-Prelog-Weg 1–5/10, 8093 Zurich, Switzerland, ^6^ETH Zurich, Department of Computer Science, Institute of Machine Learning, Universitätstrasse 6, 8092 Zurich, Switzerland, ^7^ETH Zurich, Department of Health Sciences and Technology, Otto-Stern-Weg 3, 8093 Zurich, Switzerland, ^8^ETH Zurich, Institute of Molecular Health Sciences, Otto-Stern-Weg 7, 8093 Zurich, Switzerland, ^9^ETH Zurich, NEXUS Personalized Health Technologies, John-von-Neumann-Weg 9, 8093 Zurich, Switzerland, ^10^F. Hoffmann-La Roche Ltd, Grenzacherstrasse 124, 4070 Basel, Switzerland, ^11^F. Hoffmann-La Roche Ltd, Grenzacherstrasse 124, 4070 Basel, Switzerland, ^12^Roche Diagnostics GmbH, Nonnenwald 2, 82377 Penzberg, Germany, ^13^Roche Pharmaceutical Research and Early Development, Roche Innovation Center Basel, Grenzacherstrasse 124, 4070 Basel, Switzerland, ^14^Roche Pharmaceutical Research and Early Development, Roche Innovation Center Munich, Roche Diagnostics GmbH, Nonnenwald 2, 82377 Penzberg, Germany, ^15^Roche Pharmaceutical Research and Early Development, Roche Innovation Center Zurich, Wagistrasse 10, 8952 Schlieren, Switzerland, ^16^SIB Swiss Institute of Bioinformatics, Lausanne, Switzerland, ^17^University Hospital Basel and University of Basel, Department of Biomedicine, Hebelstrasse 20, 4031 Basel, Switzerland, ^18^University Hospital Basel and University of Basel, Department of Surgery, Brustzentrum, Spitalstrasse 21, 4031 Basel, Switzerland, ^19^University Hospital Basel, Brustzentrum & Tumorzentrum, Petersgraben 4, 4031 Basel, Switzerland, ^20^University Hospital Basel, Brustzentrum, Spitalstrasse 21, 4031 Basel, Switzerland, ^21^University Hospital Basel, Department of Information- and Communication Technology, Spitalstrasse 26, 4031 Basel, Switzerland, ^22^University Hospital Basel, Gynecological Cancer Center, Spitalstrasse 21, 4031 Basel, Switzerland, ^23^University Hospital Basel, Institute of Medical Genetics and Pathology, Schönbeinstrasse 40, 4031 Basel, Switzerland, ^24^University Hospital Basel, Spitalstrasse 21/Petersgraben 4, 4031 Basel, Switzerland, ^25^University Hospital Zurich, Biomedical Informatics, Schmelzbergstrasse 26, 8006 Zurich, Switzerland, ^26^University Hospital Zurich, Clinical Trials Center, Rämistrasse 100, 8091 Zurich, Switzerland, ^27^University Hospital Zurich, Department of Dermatology, Gloriastrasse 31, 8091 Zurich, Switzerland, ^28^University Hospital Zurich, Department of Gynecology, Frauenklinikstrasse 10, 8091 Zurich, Switzerland, ^29^University Hospital Zurich, Department of Medical Oncology and Hematology, Rämistrasse 100, 8091 Zurich, Switzerland, ^30^University Hospital Zurich, Department of Pathology and Molecular Pathology, Schmelzbergstrasse 12, 8091 Zurich, Switzerland, ^31^University Hospital Zurich, Rämistrasse 100, 8091 Zurich, Switzerland, ^32^University Hospital and University of Zurich, Department of Neurology, Frauenklinikstrasse 26, 8091 Zurich, Switzerland, ^33^University of Bern, Department of BioMedical Research, Murtenstrasse 35, 3008 Bern, Switzerland, ^34^University of Zurich, Department of Quantitative Biomedicine, Winterthurerstrasse 190, 8057 Zurich, Switzerland, ^35^University of Zurich, Faculty of Medicine, Zurich, Switzerland, ^36^University of Zurich, Institute of Molecular Life Sciences, Winterthurerstrasse 190, 8057 Zurich, Switzerland, ^37^University of Zurich, Services and Support for Science IT, Winterthurerstrasse 190, 8057 Zurich, Switzerland, ^38^University of Zurich, VP Medicine, Künstlergasse 15, 8001 Zurich, Switzerland
